# Suppression of P2X4 and P2X7 by *Lactobacillus rhamnosus* vitaP1: effects on hangover symptoms

**DOI:** 10.1186/s13568-024-01685-5

**Published:** 2024-03-15

**Authors:** Jeong Eun Kwon, Woojae Hong, Hyelin Jeon, Cha Soon Kim, Hyunggun Kim, Se Chan Kang

**Affiliations:** 1 Department of Oriental Medicine Biotechnology, Kyung Hee University, Yongin, Gyeonggi 17104 Republic of Korea; 2 Department of Biomechatronic Engineering, Sungkyunkwan University, Suwon, Gyeonggi 16419 Republic of Korea; 3 Mbiometherapeutics Co., Ltd., Seongnam, Gyeonggi 13488 Republic of Korea; 4 Genencell Co., Ltd. Yongin, Gyeonggi, 16950 Republic of Korea

**Keywords:** Alcohol, Hangover, Liver improvement, P2X4, P2X7, SLC6A4

## Abstract

This study aimed to identify substances including *Lactobacillus rhamnosus* vitaP1 (KACC 92054P) that alleviate hangover-induced emotional anxiety and liver damage. The association between emotional anxiety caused by hangover and the genes P2X4, P2X7, SLC6A4 was investigated. In vitro and in vivo analyses were conducted to assess the influence of free-panica on alcohol-induced upregulated gene expression. Additionally, the concentration of AST, ALT, alcohol, and acetaldehyde in blood was measured. Free-panica, consisting of five natural products (*Phyllanthus amarus*, *Phoenix dactylifera*, *Vitis vinifera*, *Zingiber officinale*, and *Lactobacillus rhamnosus*), were evaluated for their regulatory effects on genes involved in alcohol-induced emotional anxiety and liver damage. The combination of these natural products in free-panica successfully restored emotional anxiety, and the concentration of AST, ALT, alcohol, and acetaldehyde in blood to those of the normal control group. These findings support the potential development of free-panica as a health functional food or medicinal intervention for relieving hangover symptoms and protecting liver from alcohol consumption.

## Key points

• Alcohol-induced P2X4, P2X7, and SLC6A4 gene expression was assessed.

• Mitigating alcohol-induced emotional anxiety of free-panica was assessed.

• Free-panica showed alleviating alcohol-induced emotional anxiety in vitro and in vivo.

## Introduction

Alcohol consumption is extensively prevalent worldwide. The oxidative pathway predominantly relies on the enzymatic activities of alcohol dehydrogenase (ADH) and acetaldehyde dehydrogenase (ALDH) to facilitate the sequential conversion of alcohol to acetaldehyde and subsequently to acetate (Zakhari 2006). Prolonged and excessive alcohol consumption leads to a decrease in hepatic ADH levels, which causes other detrimental reactions. Acetaldehyde, the first metabolite in alcohol oxidation, instigates a range of unpleasant hangover symptoms, including nausea, vomiting, headache, and fatigue (Mackus et al. 2020; Penning et al. 2012). Acute binge drinking is associated with hangover symptoms and potential organ damage. Hangover is generally characterized by physical and mental discomfort following alcohol consumption, encompassing dizziness, headache, fatigue, and muscle pain (Dueland 2015). These symptoms appear to result from dehydration, hormonal imbalances, dysregulated cytokine pathways, and the toxic effects of alcohol and acetaldehyde (Wang et al. 2016; Wiese et al. 2000). Notably, daily alcohol consumption exceeding 20.44 g is associated with an escalating risk of liver cancer incidence and liver disease–related mortality (Wang et al. 2016). Moreover, the protracted combination of excessive alcohol intake and environmental triggers can culminate in the establishment of habitual drinking patterns, which can be a risk factor for alcohol use disorder, an incapacitating condition afflicting a significant segment of the population (Carvalho et al. 2019). Chronic alcohol abuse and alcoholism are significant socioeconomic problems worldwide, often resulting in cognitive impairment and permanent brain damage (Crews and Vetreno 2014). Despite substantial efforts to treat hangover symptoms, liver damage, and alcohol use disorder, the quest for efficacious pharmacotherapies continues (Witkiewitz et al. 2019).

Effective pharmacological treatment is essential for addressing alcohol-induced liver damage, hangover, and alcohol use disorder, with the ultimate goal of protecting the liver, mitigating ongoing alcohol use disorder, reducing tolerance, and preventing relapse. The pharmacological management of alcohol use disorder can be divided into two phases. The initial phase primarily focuses on detoxification and managing acute liver damage and hangover symptoms, and the subsequent phase aims to prevent excessive alcohol consumption. Therefore, the demand for novel therapeutic strategies to enhance the efficacy of treatments in both phases is pressing. Recent experimental data highlight the potential of natural products as potential pharmacological interventions for hangover and alcohol use disorder (Jayawardena et al. 2017; Wang et al. 2016). Various compounds derived from plants have shown efficacy in reducing hangover symptoms, liver damage, alcohol craving, and withdrawal symptoms (Jayawardena et al. 2017; Lee et al. 2013; Penetar et al. 2015; Yamazaki et al. 2002). Notably, PartySmart, a product formulated by Himalaya using Ayurvedic principles, contains five key ingredients known for their positive effects on liver function (Pordie 2015): *Andrographis paniculata*, *Vitis vinifera*, *Emblica officinalis*, *Cichorium intybus*, and *Phyllanthus amarus* (Akbar 2011; Nassiri-Asl and Hosseinzadeh 2016; Patel et al. 2011; Street et al. 2013; Variya et al. 2016). PartySmart enhances metabolism by increasing the activity of ADH and ALDH without causing adverse effects (Venkataranganna et al. 2008). It ameliorates alcohol-induced liver injury by preventing disruptions in cell membranes, reducing oxidative stress through free radical scavenging and antioxidant activity, and restoring intracellular redox status to normal levels (Gopumadhavan et al. 2008). However, further investigations are required to validate the effectiveness of PartySmart in preventing and treating hangover symptoms such as emotional instability and alcohol use disorder.

Free-panica consists of *Phyllanthus amarus*, *Phoenix dactylifera*, *Vitis vinifera*, *Zingibe officinale*, *Lactobacillus rhamnosus*. *P. amarus* and *V. vinifera*, which are included in PartySmart, are well-known for their effectiveness in improving liver function. Widely distributed globally, *P. dactylifera* is popular as a principal dietary staple, particularly in in Arabian countries (GIF 2008). *P. dactylifera* has been used to address a spectrum of infectious diseases and cancers in traditional remedies (Duke 1992). Moreover, recent studies have underscored its effectiveness in mitigating oxidative stress-induced liver damage (Saafi et al. 2011). *Z. officinale* (ginger) has a long history as a culinary spice for mora than a thousand years (Bartley and Jacobs 2000). Studies revealed *Z. officinale* as a detoxifying agent mitigating the adverse effects of alcohol abuse, a hepatic anticancer agent suggesting a role in countering hepatic malignancies, and a nutraceutical agent demonstrating promise in addressing liver fibrosis (Shati and Elsaid 2009; Habib et al. 2008; Motawi et al. 2011). *L. rhamnosus* has been recognized for its beneficial effects, particularly in mitigating alanine aminotransferase (ALT) levels, liver hepatitis, steatosis in both non-alcoholic fatty liver disease (NAFLD) and alcoholic liver disease (ALD) (Vajro et al. 2011; Ritze et al. 2014).

Purinergic P2X4 and P2X7, along with the serotonin transporter-linked polymorphic region (5-HTTLPR) of the serotonin transporter gene (SLC6A4), have previously been implicated in alcohol-related risks. P2X4Rs and P2X7Rs belong to the P2X superfamily of ionotropic receptors, which are activated by adenosine-5’-triphosphate (ATP) (North 2016). Accumulating evidence suggests that P2X4s and P2X7s play crucial roles in mediating ethanol-induced responses in microglia (Asatryan et al. 2018). P2X4Rs are highly expressed in the central nervous system (CNS) and are responsive to low concentrations of intoxicating ethanol (Suurvali et al. 2017). A genetic meta-analysis identified the P2X4 gene as a candidate gene for innate alcohol intake and preference (Suurvali et al. 2017). Notably, a reduction in P2X4 expression has been linked to an increase ethanol consumption and preference (Khoja et al. 2018). Pharmacological inhibition studies have demonstrated the significant involvement of P2X7 in generating inflammatory responses during various CNS pathologies. P2X7 inhibition effectively reduces the inflammatory response in the brain and liver that is induced by chronic alcohol consumption (Freire et al. 2019). Ethanol exerts inhibitory effects on P2X4 (Franklin et al. 2015), but P2X7 channel activity remains unaffected. Furthermore, ethanol suppresses P2X4-mediated microglial migration while enhancing pore formation in P2X7 (Asatryan et al. 2018). Ethanol also induces the protein expression of both P2X receptor subtypes (Asatryan et al. 2018). Among the initial candidate genes explored for their potential association with the risk of alcohol use disorder, SLC6A4 was prioritized (Cope et al. 2017). Variations in SLC6A4 have been linked to traits such as anxiety and depression, which are also associated with alcohol use disorder (Thompson and Kenna 2016). Ethanol ingestion leads to the downregulation of SLC6A4 expression. Therefore, those three genes were selected as targets for evaluating alcohol-related risk (Madaan et al. 2022).

The objective of this study was to explore the potential of *Lactobacillus rhanmnosus* (*L. rhamnosus*) vitaP1 and free-panica in mitigating, preventing, and treating alcohol-induced hangover symptoms, liver damage, and alcohol use disorder. Ethanol-induced in vitro and in vivo models were employed to evaluate the effects of *L. rhamnosus* vitaP1 and free-panica on ethanol-induced symptoms.

## Materials and methods

A series of experiments were conducted to investigate the effects of *L. rhamnosus* vitaP1 (KACC 92054P), deposited in Korean Agricultural Culture Collection, Wanju, Republic of Korea, and free-panica on ethanol-induced symptoms. Subsequently, a combination of hepatoprotective and liver function-enhancing plants and *L. rhamnosus* vitaP1 was formulated as free-panica and administered to ethanol-induced mice to evaluate the potential of free-panica to relieve hangover symptoms, protect against liver damage, and prevent alcohol use disorder.

In the first experiment, BV2 cells were used to determine the threshold ethanol concentration that produced a significant increase in P2X4 and P2X7 gene expression. Subsequently, we evaluated the inhibitory effects of extracts from the primary components of the Ayurvedic PartySmart ingredients on the ethanol-induced upregulation of P2X4 and P2X7 gene expression. In the second experiment, the ability of *L. rhamnosus* vitaP1 and free-panica to attenuate the increased expression of the P2X4 and P2X7 genes was examined using ethanol-induced BV2 cells. The third experiment involved measuring aspartate aminotransferase (AST) and alanine aminotransferase (ALT) levels in blood serum and the expression levels of P2X4, P2X7, and SLC6A4 genes in hippocampal tissue from ethanol-induced mouse models treated with an oral administration of *L. rhamnosus* vitaP1 and free-panica. Lastly, the influence of free-panica, a combination of four plant extracts and *L. rhamnosus* vitaP1, was assessed in an ethanol-induced mouse model. A comprehensive assessment was conducted by quantifying alcohol, acetaldehyde, AST, and ALT levels in blood serum and the expression of the P2X4, P2X7, and SLC6A4 genes in hippocampal tissue from the mice.

### Preparation of ***L. rhamnosus*** vitaP1

*Lactobacillus rhamnosus* VitaP1 was cultured with the following medium composition: 2% hydrolyzed soybean protein (HSP349, Tatua Co-operative Dairy Company Limited, New Zealand), 2% yeast extract (NuCel 584MG, Guangxi Yipinxian Biotechnology Co., Ltd, China), 0.25% potassium phosphate, 2.5% D-glucose, 0.1% L-cysteine, and 0.4% sodium acetate. The medium was sterilized before use, and after the *L. rhamnosus* VitaP1 was inoculated at 0.1%, it was cultured at 37℃ for 72 h. After culture, pellets (microorganisms) and supernatants were separated through centrifugation and freeze-dried and powdered, respectively.

### Extraction of the dried plants and preparation of free-panica

*A. paniculata, C. intybus*, *P. tenellus, V. vinifera, P. dactylifera*, and *P. emblica* were purchased from a traditional market (Seoul, Korea). The extracts used in the experiment were prepared from dried plants (*A. paniculata, C. intybus*, *P. tenellus, V. vinifera, P. dactylifera*, and *P. emblica*) with boiling water for 4 to 6 h, then concentrated to 30 brix under reduced pressure. *Zingiber officinale* Roscoe was purchased from Wanju-gun (Korea) in April 2019. The extract was processed in the following manner. The ginger was washed three times with distilled water to remove sand and dust, and then the washed ginger was steamed under the following conditions: 2–2.5 kgf/cm^2^, 97 °C, 2 h. Golden ginger (GG) was obtained by extracting the steamed ginger with fifteen-fold of 70% ethanol (v/v) for 15 h at 85 °C, 1.5 kg/cm^2^. The GG was filtered through 60 mesh and concentrated at -650 mmHg, 55 °C. Then the extract was spray-dried to obtain a powder that was stored at -20 °C until use. The free-panica consisted of 30% GGE03, 10% *L. rhamnosus* VitaP1, 10% *V. vinifera* extract, 20% *P. dactylifera* extract, and 30% *P. emblica* extract.

### BV2 cell culture

BV2 microglial cells were purchased from the Korean Cell Line Bank. The cells were grown in complete DMEM with penicillin/streptomycin (10,000 U/mL) and 10% fetal bovine serum (Thermo Fisher Scientific (MA, USA)). The culture was maintained at 37℃ in a 5% CO_2_ atmosphere.

### Determining P2X4, P2X7, and SLC6A4 gene expression by quantitative RT-PCR

To determine how much ethanol was needed to increase the expression of the P2X4 and P2X7 genes, BV2 cells were treated with various ethanol concentrations for 12 h, and mRNA was extracted to measure the expression of the P2X4 and P2X7 genes.

**Table 1:** The sequences used for quantitative real time-PCR

Gene	Forward	Reverse
P2X4	5’-TTGGCTCTGGCTTGCGCTC-3’	5’-TCTCCGGAAAGACCCTGCTCG-3’
P2X7	5’-TCTTCGTGATGACAAACTTTCTCAA-3’	5’-TCTCCACTGGGCACCAACTC–3’
SLC6A4	5’-GTTGATGCTGCGGCTCAGATCT-3’	5’-GAAGCTCGTCATGCAGTTCACC-3’
β-actin	5’-CCTGACCCTGAAGTACCCCA-3’	5’-CGTCATGCAGCTCATAGCTC-3’

### Hangover relief effect after acute alcohol administration

Six-week-old C57BL/6 mice with a weight of 25 ± 2 g were purchased from RaonBio Co., Ltd (Yongin, Korea) and kept on a 12-hour light and dark cycle at 25℃ and 40–60% humidity. The mice were allowed to acclimate for one week and were then divided into 10 mice per group.

The procedures for the experiments and animal care protocol (Approval number: KHGASP-21-359) were approved by the Internal Animal Care and Use Committee of Kyung Hee University. The experimental procedures followed the National Institutes of Health guidelines for the care and use of laboratory animals.

To eliminate differences in alcohol absorption and decomposition caused by diet, each mouse was given the test substance by oral administration after fasting for 12 h; 30 min later, 20% ethanol (5 g/kg) was orally administered. Blood was collected 1 h, 3 h, and 5 h after alcohol administration and centrifuged at 14,000 rpm for 20 min to separate the serum. The separated serum was used to measure the concentrations of alcohol, acetaldehyde, ALT, and AST.

After hippocampal tissue from each mouse was extracted, it was cut into small pieces, homogenized with chloroform, and the supernatant was separated. The supernatant was reacted with isopropanol to obtain pellets, which were washed with 70% ethanol, diluted with sterile distilled water, and quantified. cDNA synthesis was performed using a PrimeScript™ 1st strand cDNA synthesis kit (Takara, Japan). The expression of the P2X4, P2X7, and SLC6A4 genes at the mRNA level in the hippocampal tissue was evaluated using qRT-PCR (Table 1).

### Biochemical analysis

ALT and AST levels in serum were measured using kits from Asan Pharm (Seoul, Korea). Serum (20 μM) was mixed with ALT and AST substrate solutions and reacted at 37℃ for 60 min and 30 min, respectively. Then, 100 μL of 2,4-dinitrophenylhydrazine was added and allowed to react at room temperature for 20 min. After 1 ml of 0.4 N NaOH was added to stop the enzyme reaction, the absorbance was measured at 505 nm using a multi reader (Tecan, Switzerland).

To determine serum alcohol content, an alcohol assay kit (Cell Biolabs Inc., CA, USA) using the enzyme colorimetric principle was used, and absorbance was measured at 570 nm. The level of acetaldehyde in the serum was measured at 340 nm using an acetaldehyde assay kit (Roche Co., Ltd, Basel, Switzerland).

### Statistical analysis

Data were evaluated using GraphPad Prism (version 5.0). All data are expressed as the mean ± standard error (SE) of 3 or 4 independent experiments, and the significance of differences between groups was calculated using a one-way ANOVA followed by Turkey’s post hoc means comparison test or Student’s t-test.

## Results

### Effects of PartySmart compounds on P2X4 and P2X7 gene expression in vitro

**Fig. 1:**
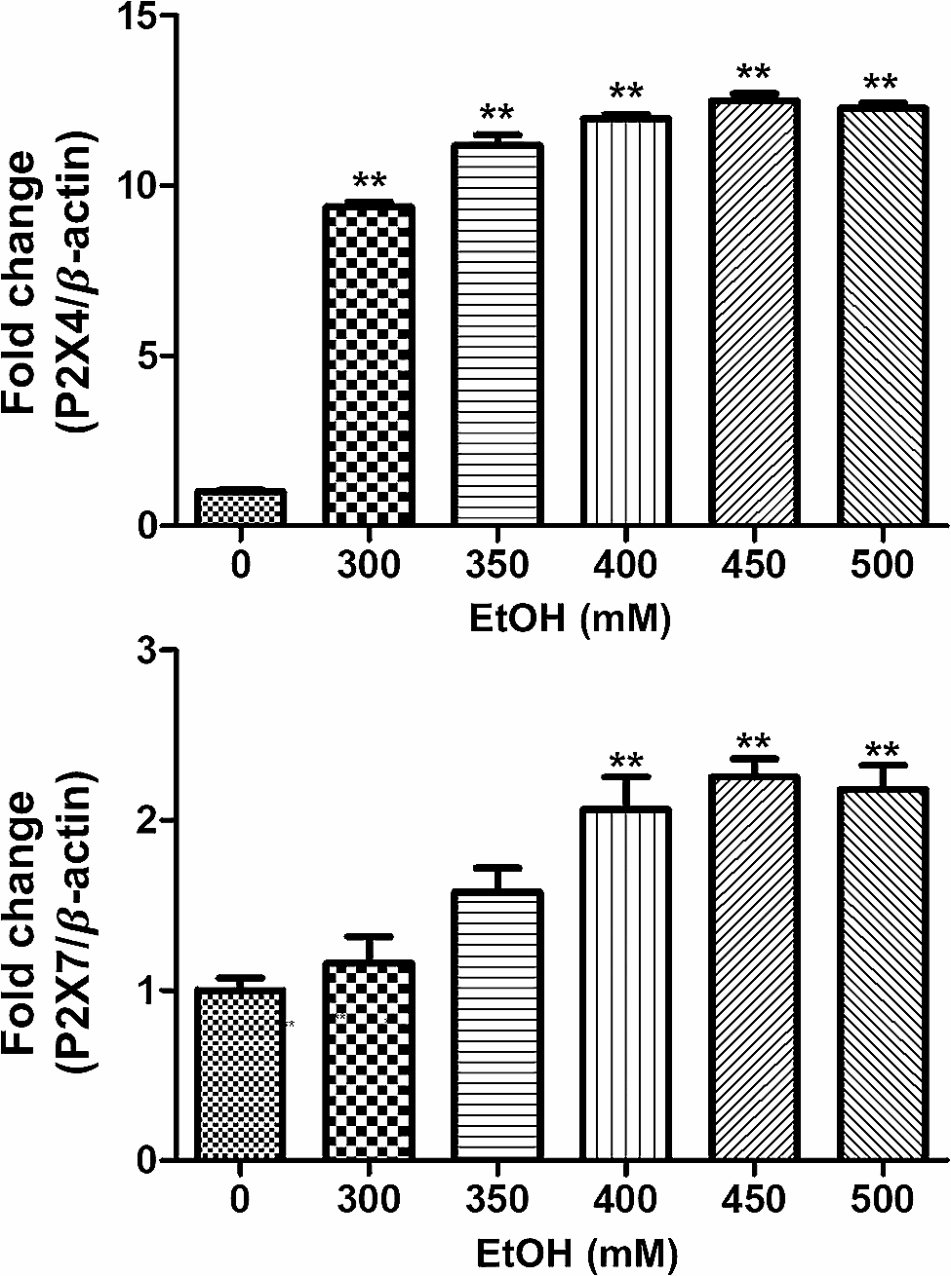
Effects of ethanol on the mRNA expression of the P2X4 and P2X7 genes in BV2 cells. Ethanol increased the mRNA levels of the P2X4 and P2X7 genes. *p < 0.05 and **p < 0.01 compared with control. Data are shown as mean ± SEM (*n* = 3)

**Fig. 2:**
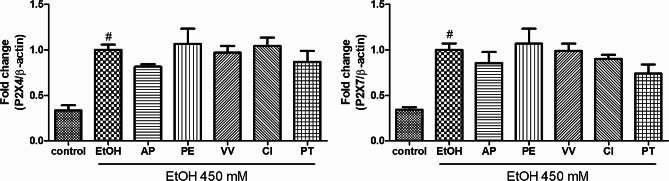
Effects of the PartySmart composition on the mRNA expression of the P2X4 and P2X7 genes in BV2 cells. ^#^*p* < 0.05 compared with control. There was no statistical difference found between the ethanol-treated control and other treatment groups. Data are shown as mean ± SEM (*n* = 3). AP: *Andrographis paniculata*, VV: *Vitis vinifera*, PE: *Emblica officinalis*, CI: *Cichorium intybus*, and PT: *Phyllanthus amarus*

To determine the threshold ethanol concentration at which P2X4 and P2X7 gene expression was significantly upregulated, BV2 cells were exposed to various ethanol concentrations. A large increase in P2X4 and P2X7 gene expression was observed at an ethanol concentration of 450 mM (Fig. 1). Subsequently, we investigated the inhibitory effects of extracts obtained from the five primary Ayurvedic PartySmart ingredients on the upregulation of P2X4 and P2X7 gene expression induced by 450 mM ethanol. While *A. paniculata* and *P. amarus* extracts tended to inhibit elevated expression of the P2X4 (*p* = 0.0506 and *p* = 0.3856, respectively) and P2X7 (*p* = 0.3837 and *p* = 0.1457, respectively) genes, there was no significant differences found with respect to the ethanol-treated cells (Fig. 2).

### Effects of ***L. rhamnosus*** vitaP1 and free-panica on P2X4 and P2X7 gene expression in vitro

We tested how well 9 microorganisms reported to improve liver function (*Bifidobacterium bifidum* MBT04 (Wang et al. 2022), *B. longum* MBT10 (Xie and Halegoua-DeMarzio 2019), *Clostridium butyricum* BM-02 (Liu et al. 2017), *L. lactis* MBT02 (Delgado-Venegas et al. 2021; Yu et al. 2021), *L. casei* SEquo1028 (Li et al. 2021), *Pediococcus pentosaceus* SEQ0315 (Yu et al. 2021), *Leuconostoc mesenteroides* MBT17 (Castro-Rodriguez et al. 2020), *L. rhamnosus* VitaP1 (Jeong et al. 2022; Liu et al. 2020), *L. sakei* SEQvitaP (Nguyen et al. 2022) protected BV2 cells damaged by ethanol. Among those 9 species, BV2 cells damaged by ethanol recovered after exposure to *C. butyricum* BM-02 and *L. rhamnosus* VitaP1 (data not shown). Between them, *L. rhamnosus* VitaP1 offered excellent BV2 cell protection, so the further experiments were conducted using *L. rhamnosus* VitaP1.

**Fig. 3:**
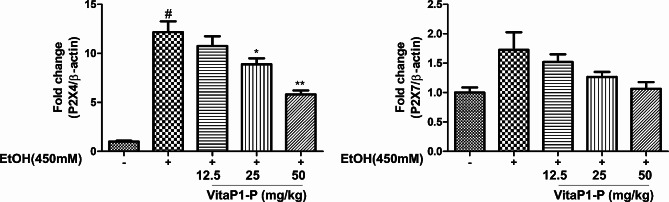
Effects of *Lactobacillus rhamnosus* VitaP1 on the mRNA expression of the P2X4 and P2X7 genes in BV2 cells. *L. rhamnosus* VitaP1 inhibited the mRNA levels of the P2X4 and P2X7 genes. VitaP1-P: *L. rhamnosus* VitaP1. ^#^*p* < 0.05 compared with control. *p < 0.05 and **p < 0.01 compared with ethanol-treated control. Data are shown as mean ± SEM (*n* = 3)

To assess the ability of *L. rhamnosus* vitaP1 to mitigate the enhanced expression of the P2X4 and P2X7 genes, BV2 cells were exposed to 450 mM ethanol and subsequently treated with *L. rhamnosus* vitaP1. Strikingly, *L. rhamnosus* vitaP1 revealed a substantial inhibitory effect on the elevated expression of the P2X4 and P2X7 genes at a concentration of 50 μg/mL (Fig. 3).

### Effects of ***L. rhamnosus*** vitaP1 on AST and ALT levels and P2RX4, P2RX7, and SLC6A4 gene expression in vivo

**Fig. 4:**
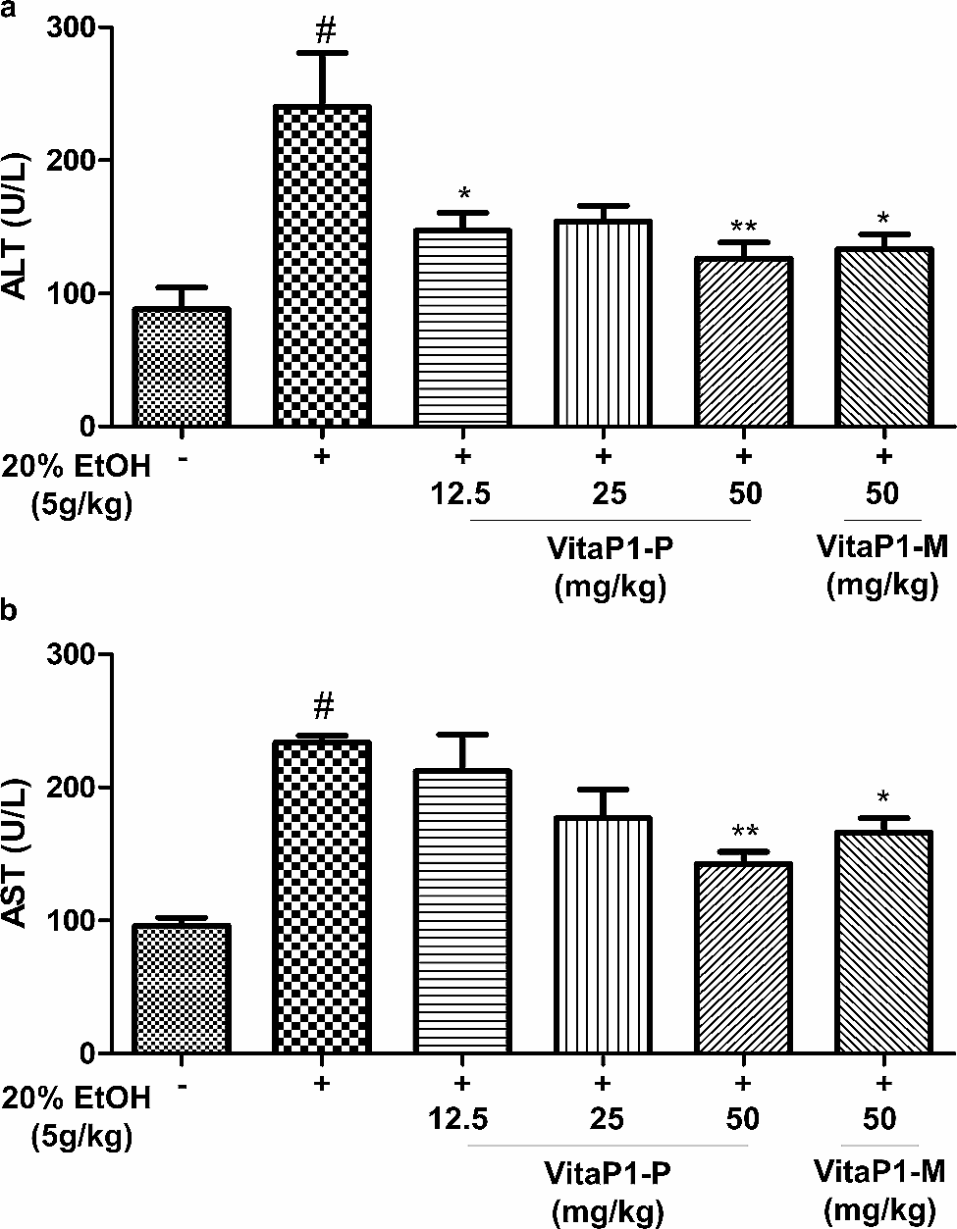
Effects of *Lactobacillus rhamnosus* VitaP1 on the serum (**A**) ALT and (**B**) AST concentrations in mice 3 h after an oral administration of ethanol. ^#^*p* < 0.05 compared with control. *p < 0.05 and **p < 0.01 compared with ethanol-treated control. Data are shown as mean ± SEM (*n* = 3). VitaP1-P: *L. rhamnosus* VitaP1 strain powder, VitaP1-M: *L. rhamnosus* VitaP1 culture

After an oral administration of *L. rhamnosus* vitaP1 strain powder (VitaP1-P, 12.5, 25, and 50 mg/kg) or *L. rhamnosus* vitaP1 culture powder (VitaP1-M, 50 mg/kg), mice treated with 450 mM ethanol were comprehensively assessed. The AST levels displayed dose-dependent attenuation upon treatment with VitaP1-P. A considerable decrease in AST levels was found at a dose of 50 mg/kg for both VitaP1-P and VitaP1-M (Fig. 4).

**Fig. 5:**
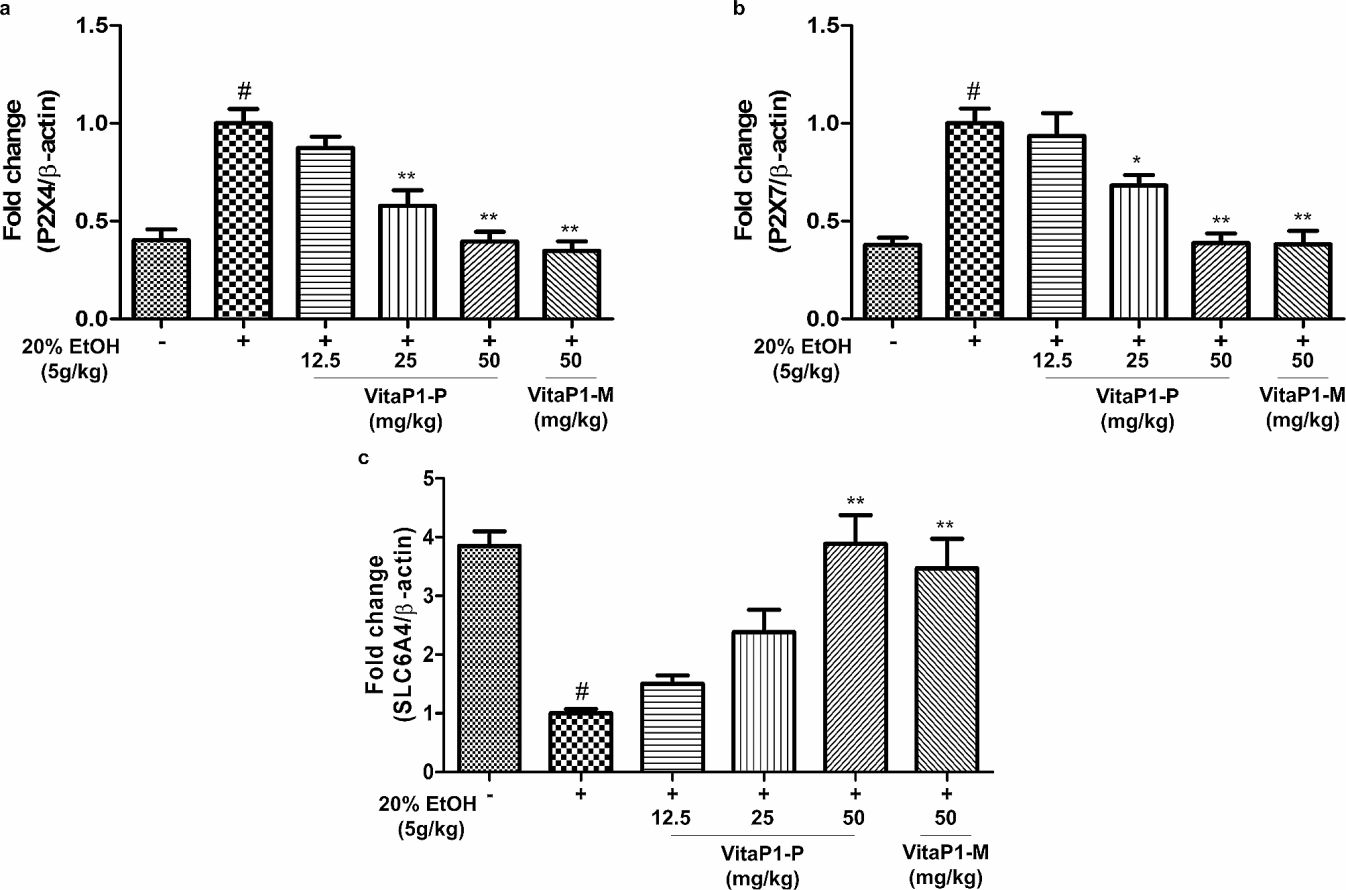
Effects of *Lactobacillus rhamnosus* VitaP1 on the mRNA expression of the P2X4, P2X7, and SLC6A4 genes in hippocampal tissue. *L. rhamnosus* VitaP1 inhibited the mRNA levels of the P2X4 and P2X7 genes. ^#^*p* < 0.05 compared with control. *p < 0.05 and **p < 0.01 compared with the ethanol-treated control. Data are shown as mean ± SEM (*n* = 3). VitaP1-P: *L. rhamnosus* VitaP1 strain powder, VitaP1-M: *L. rhamnosus* VitaP1 culture

Similarly, a significant improvement in ALT levels was observed upon an administration of VitaP1-P or VitaP1-M at a dose of 50 mg/kg (Fig. 4). Treatment with 450 mM ethanol induced an increase in the expression of the P2X4 and P2X7 genes, and oral administration of VitaP1-P exhibited suppressive effects on that increase in a dose-dependent manner. Notably, at a dose of 50 mg/kg, both VitaP1-P and VitaP1-M restored the expression of the P2X4 and P2X7 genes to levels comparable to those observed under normal conditions. In contrast, the expression of the SLC6A4 gene was reduced by 450 mM ethanol, and oral administration of VitaP1-P produced a dose-dependent restoration. Intriguingly, ethanol-induced mice treated with a dose of 50 mg/kg of VitaP1-P or VitaP1-M exhibited gene expressions similar to those observed under normal conditions (Fig. 5).

### Effects of free-panica on alcohol, acetaldehyde, AST, and ALT levels and P2X4, P2X7, and SLC6A4 gene expression in vivo

**Fig. 6:**
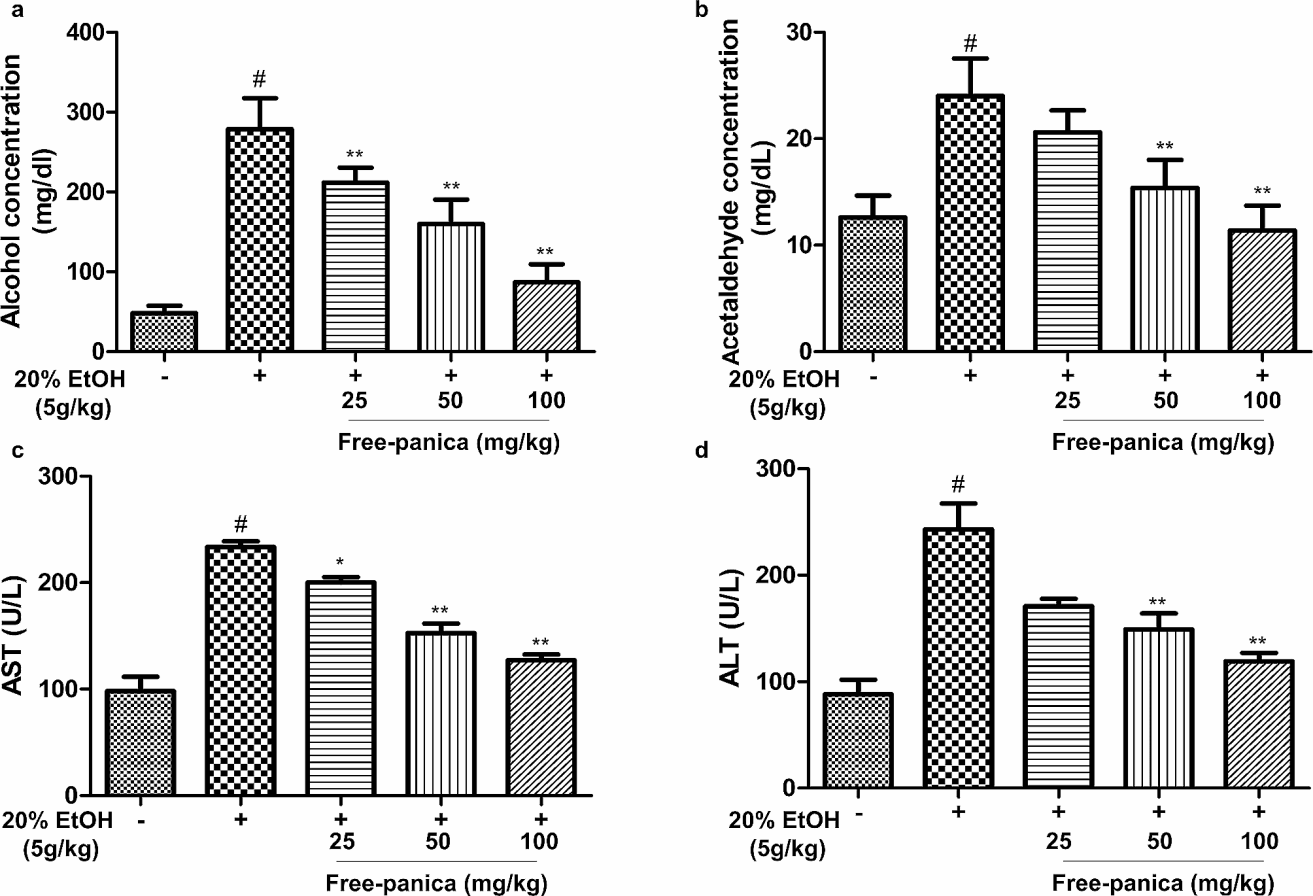
Effects of free-panica on (**A**) serum alcohol, (**B**) acetaldehyde, (**C**) ALT, and (**D**) AST concentrations in mice 3 h after oral administration of ethanol. Free-panica reduced both alcohol and acetaldehyde concentrations as well as the levels of AST and ALT in the blood serum. ^#^*p* < 0.05 compared with control. *p < 0.05 and **p < 0.01 compared with ethanol-treated control. Data are shown as mean ± SEM (*n* = 3)

**Fig. 7:**
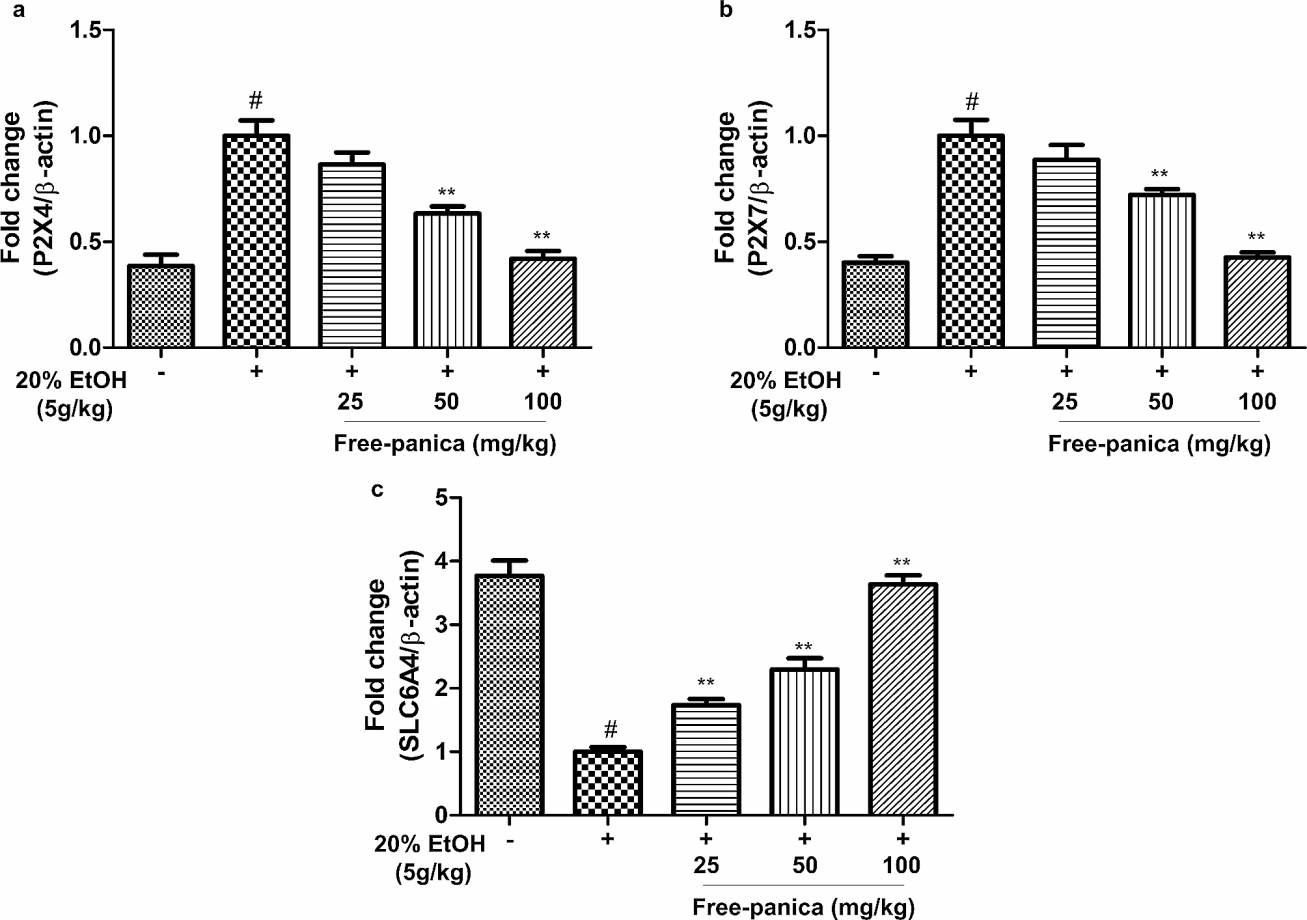
Effects of free-panica on the mRNA expression of the P2X4, P2X7, and SLC6A4 genes in hippocampal tissue. Free-panica inhibited the mRNA levels of the P2X4 and P2X7 genes. ^#^*p* < 0.05 compared with control. *p < 0.05 and **p < 0.01 compared with the ethanol-treated control. Data are shown as mean ± SEM (*n* = 3)

This experimental study investigated the effects of free-panica in ethanol-induced mice. Blood serum alcohol and acetaldehyde concentrations and AST and ALT levels were assessed. Oral administration of the mixture had a dose-dependent restorative effect, successfully mitigating all ethanol-induced changes. The oral administration of the mixture at a dose of 100 mg/kg substantially reduced both alcohol and acetaldehyde concentrations in the blood serum. Furthermore, the levels of AST and ALT in blood serum exhibited a large amelioration at the same dose (Fig. 6). Importantly, the expression levels of the three key genes (P2RX4, P2RX7, and SLC6A4) were also effectively restored to their normal levels upon oral administration of the free-panica mixture at 100 mg/kg (Fig. 7).

## Discussion

This study investigated the potential of *L. rhamnosus* vitaP1 and free-panica, a synergistic mixture of GG, *L. rhamnosus* VitaP1, *V. vinifera* extract, *P. dactylifera* extract, and *P. emblica* extract, to mitigate alcohol-induced hangover symptoms, liver damage, and alcohol use disorder. Ethanol-induced in vitro and in vivo models were used to evaluate the effects of *L. rhamnosus* vitaP1 and free-panica. Multiple parameters, including the gene expression levels of P2X4, P2X7, and SLC6A4 and the levels of biochemical markers, were assessed to determine the efficacy of the interventions.

Alcohol hangover is an unpleasant state caused by acetaldehyde, which is produced by partial oxidation of ethanol. Acetaldehyde stimulates the vagus nerve and afferent nerve fibers in sympathetic nerves, causing hangover symptoms such as nausea, dizziness, pupil enlargement, increased heart rate, and accelerated respiration. Many studies on hangover symptoms have utilized ethanol doses ranging between 0.5 and 1 g/kg (Nagai et al. 2001; Choi et al. 2023). Nagai et al. (2001) have reported that a significant change in hemorheologic factors occurs when ethanol is administered at 1 g/kg (See the captured contents below). In our study, 20% ethanol was administered at 5 g/kg. Subsequently, we monitored acetaldehyde concentrations in mice at hourly intervals.

Recent advancements suggest that chronic alcohol consumption can trigger neurodegenerative brain damage, primarily through persistent neuroinflammation. Investigations employing diverse animal models of ethanol exposure demonstrated a range of responses in microglia, including partial or full activation, as well as microglial apoptosis. Furthermore, enhancements in pro-inflammatory mediators evident in animal models chronically exposed to ethanol, as well as in post-mortem alcoholic human brain specimens, have been correlated with alterations in glial cells, particularly affecting microglial dynamics.

The impact of P2X4, P2X7, and SLC6A4 genes on alcohol-related risks has been extensively studied. Ethanol-induced expression of P2X4 and P2X7 receptors is crucial for the microglial responses to ethanol. P2X4 receptors are implicated in ethanol intake and preference, while P2X7 receptors contribute to pro-inflammatory responses induced by ethanol (Freire et al. 2019; Khoja et al. 2018). Variations in SLC6A4 are connected to traits such as anxiety and depression, which are also related to alcohol use disorder (Cope et al. 2017). Ethanol consumption downregulates SLC6A4 expression. Therefore, assessing the gene expressions of P2X4, P2X7, and SLC6A4 is a suitable way to diagnose hangover-related emotional anxiety and alcohol use disorder. The release of AST and ALT into the bloodstream and subsequent increase in their serum concentrations and activities indicate liver tissue damage. Therefore, measuring AST and ALT levels in serum is an effective method for diagnosing liver damage (Szabo et al. 2011).

Himalaya’s PartySmart, an Ayurvedic product, contains five key ingredients: *A. paniculata*, *V. vinifera*, *E. officinalis*, *C. intybus*, and *P. amarus*. *A. paniculata* is recognized for its medicinal properties and potential liver protection (Akbar 2011). *Vitis vinifera*, commonly known as grape, exhibits antioxidant activity and holds promise in promoting liver health (Nassiri-Asl and Hosseinzadeh 2016). *E. officinalis*, or Indian gooseberry, possesses hepatoprotective properties and a rich antioxidant profile (Variya et al. 2016). *Cichorium intybus*, also known as chicory, has garnered scientific attention for its hepatoprotective effects and potential in maintaining liver function (Street et al. 2013). *P. amarus*, a traditional medicinal plant, has demonstrated hepatoprotective properties and could aid in mitigating liver damage (Patel et al. 2011). To explore their efficacy in addressing hangover symptoms, including emotional instability and alcohol use disorder, extracts of those five plants were evaluated for their ability to suppress the ethanol-induced upregulation of P2X4 and P2X7 gene expression in BV2 cells. *A. paniculata* and *P. amarus* demonstrated suppressive effects on P2X4 and P2X7 gene expression. Therefore, PartySmart has the potential to mitigate hangover symptoms, particularly emotional instability.

*Lactobacillus* spp. possess beneficial properties that can improve gastrointestinal disease, allergies, and liver diseases through diverse mechanisms. These mechanisms encompass the production of metabolites with pathogen-suppressing effects, immune regulatory capabilities, and modulation of intestinal microbial groups (Hojsak et al. 2010; Joo et al. 2011; Kwon et al. 2010; Yonekura et al. 2009). Previous research has demonstrated the efficacy of *L. rhamnosus* in the prevention and treatment of liver disease in both patients and animal models (Mantegazza et al. 2018; Wang et al. 2013). Furthermore, *L. rhamnosus* has exhibited potential in reducing liver fibrosis in rats (Hammes et al. 2017). Considering the positive influence of *L. rhamnosus* on liver function, we hypothesized its potential impact on hangover relief. To assess this, BV2 cells were exposed to ethanol-induced damage, followed by treatment with a microbial culture solution containing *L. rhamnosus* to evaluate its capacity to restore the proliferation of damaged BV2 cells.

The inhibitory effects of *L. rhamnosus* vitaP1 and free-panica on ethanol-induced upregulation of P2X4 and P2X7 gene expression in BV2 cells were investigated. The efficacy of these probiotics in mitigating hangover symptoms, including emotional instability and alcohol use disorder, was examined using mice models of ethanol-induced acute liver failure. The evaluation encompassed the measurement of AST and ALT levels in blood serum and the analysis of P2X4, P2X7, and SLC6A4 gene expression in hippocampal tissue. *L. rhamnosus* vitaP1 exhibited a remarkable ability to suppress the upregulated expression of P2X4 and P2X7 genes in BV2 cells at a concentration of 50 μg/mL. The administration of *L. rhamnosus* vitaP1 and free-panica at a dose of 50 mg/kg demonstrated a considerable improvement in AST and ALT levels. *L. rhamnosus* vitaP1 and free-panica restored the expression of P2X4 and P2X7 genes to levels comparable to those observed under normal conditions. Conversely, the expression of the SLC6A4 gene was reduced by 450 mM ethanol. Oral administration of both *L. rhamnosus* vitaP1 and free-panica restored SLC6A4 gene expression to levels similar to those observed under normal physiological conditions. These findings indicate the ability of *L. rhamnosus* vitaP1 and free-panica to alleviate hangover symptoms, preserve liver integrity, and prevent the onset of alcohol use disorder.

Enhancing liver function is considered a promising strategy to alleviate alcohol-induced hangovers, and the free-panica formulation consists of a blend of natural products known for their hepatoprotective properties. Ginger has been extensively reported to have antioxidant, anti-inflammatory, insecticidal, and tocolytic effects. Ginger has demonstrated efficacy in the treatment and prevention of gastritis, gastric ulcers, arthritis, and metabolic diseases within the gastrointestinal tract. *Vitis vinifera*, commonly known as grapevine, is native to central Europe and southwestern Asia, with its native range spanning from Morocco and Spain in the west to southern Germany in the north, and northern Iran in the east. The dried fruit possesses demulcent, cooling, sweet, laxative, and stomachic properties, and has been traditionally used to alleviate thirst and coughs. Additionally, it has been employed in the treatment of liver disorders, uterine tumors, and cancer (Lee et al. 2003; Sharma et al. 2012). *Phoenix dactylifera* holds substantial dietary significance in numerous global regions, particularly in Arabian countries. *P. dactylifera* features prominently in remedies for diverse infectious diseases and cancer (Duke 1992). Recent studies have indicated its strong antioxidant effect and ability to inhibit oxidative stress in the liver (Allaith 2008; Saafi et al. 2009, 2011). *Phyllanthus emblica*, widely distributed in subtropical and tropical regions such as Taiwan, China, and India, plays a pivotal role in the treatment of liver disease (Bagalkotkar et al. 2006; Krishnaveni and Mirunalini 2010).

The efficacy of a combination of microorganisms and four plant extracts in alleviating hangover symptoms, providing hepatoprotection, and preventing alcohol use disorder was investigated using an ethanol-induced acute liver failure mouse model. The administration of the mixture at a dose of 100 mg/kg markedly decreased alcohol and acetaldehyde concentrations in blood serum, substantiating its therapeutic potential. Moreover, the same dose exhibited notable efficacy in mitigating AST and ALT levels in blood serum. Oral administration of the mixture at the same dose significantly restored the expression levels of the P2X4, P2X7, and SLC6A4 genes to their normal levels. These findings provide compelling evidence that free-panica could alleviate hangover symptoms, preserve liver integrity, and potentially prevent the onset of alcohol use disorder.

Elevated adenine levels resulting from ATP depletion due to alcohol consumption led to emotional symptoms such as lethargy and anxiety, accompanied by altered expression of P2X4, P2X7, and SLC6A4 genes. Five natural products recognized for their ability to modulate genes associated with alcohol-induced emotional anxiety were integrated to create a formulation called free-panica. Our findings confirmed that free-panica not only mitigated alcohol-induced emotional anxiety but also restored blood alcohol concentration, acetaldehyde levels, and gene expression to their normal states. Consequently, our findings validate the potential value of free-panica as a health functional food or medicine for effectively alleviating hangover symptoms.

## Data Availability

The data that support the findings of this study could be available by the corresponding authors, upon reasonable request.
